# 2-Chloro-3,7,8-tribromodibenzofuran as a new environmental pollutant inducing atypical ultrasonic vocalization in infant mice

**DOI:** 10.1093/toxres/tfad069

**Published:** 2023-08-23

**Authors:** Eiki Kimura, Go Suzuki, Naoto Uramaru, Masaki Kakeyama, Fumihiko Maekawa

**Affiliations:** Health and Environmental Risk Research Division, National Institute for Environmental Studies, Tsukuba 305-8506, Japan; Japan Society for the Promotion of Science, Tokyo 102-0083, Japan; Department of Environmental Health, University of Fukui School of Medical Sciences, Fukui 910-1193, Japan; Material Cycles Division, National Institute for Environmental Studies, Tsukuba 305-8506, Japan; Division of Pharmaceutical Health Biosciences, Nihon Pharmaceutical University, Saitama 362-0806, Japan; Faculty of Human Sciences, Waseda University, Saitama 359-1192, Japan; Health and Environmental Risk Research Division, National Institute for Environmental Studies, Tsukuba 305-8506, Japan

**Keywords:** developmental neurotoxicity, mixed polybrominated/chlorinated dibenzofuran, mouse, ultrasonic vocalization

## Abstract

Epidemiological and experimental studies indicate that maternal exposure to environmental pollutants impairs the cognitive and motor functions of offspring in humans and laboratory animals. Infant ultrasonic vocalizations (USVs), the communicative behavior of pups toward caregivers, are impaired in rodent models of neurodevelopmental disorders, suggesting a useful method to evaluate the developmental neurotoxicity of environmental pollutants. Therefore, we investigated USVs emitted by mouse pups of dams exposed to 2-chloro-3,7,8-tribromodibenzofuran (TeXDF) and 1,2,3,7,8-pentabromodibenzofuran (PeBDF), which are detected in the actual environment. The USV duration and number in the pups born to dams administered with TeXDF 40 μg/kg body weight (b.w.), but not 8 μg/kg b.w., on gestational day (GD) 12.5, were significantly lower than those in the corresponding pups on postnatal days 3–9. Conversely, there was no statistical change in the USVs emitted by the pups of dams administered with PeBDF 35 or 175 μg/kg b.w. on GD 12.5. To examine whether maternal exposure leads to behavioral impairments in adulthood, we analyzed exploratory behaviors in a novel environment using IntelliCage, a fully automated testing apparatus for group-housed mice. Neither TeXDF nor PeBDF exposure induced significant differences in offspring exploration. Considered together, our findings revealed that TeXDF induces atypical USV emission in infant mice, suggesting the importance of further studies on the risk assessment of mixed brominated/chlorinated dibenzo-*p*-dioxins and dibenzofurans.

## Introduction

Early-life exposure to environmental pollutants, such as metals and organic compounds, adversely affects neurodevelopment, leading to abnormalities in the brain structure, activity, and function.^1^ Epidemiological studies have shown impairment of cognitive and motor development in children exposed to polychlorinated dibenzo-*p*-dioxins/dibenzofurans (PCDD/Fs) through maternal breast milk.^2–4^ In animal experiments, rodent offspring born to dams administered with 2,3,7,8-tetrachlorodibenzo-*p*-dioxin (TeCDD), the most toxic congener in the group of PCDD/Fs,^5^ exhibit higher concentrations of TeCDD in the brain in a dose-dependent manner,^6^ and behavioral abnormalities.^7^^,^^8^ Polybrominated dibenzo-*p*-dioxins/dibenzofurans (PBDD/Fs), by-products of brominated flame retardants widely used in commodities (i.e. plastics, electrical applications, and vehicles),^9^ have been detected in food and biological samples.^10^^,^^11^ Animal experiments have revealed developmental toxicity in fetal mice exposed to 2,3,7,8-tetrabromodibenzo-*p*-dioxin (TeBDD) or 2,3,7,8-tetrabromodibenzofuran (TeBDF).^12^ To evaluate the risks of these chemicals, the toxic potencies of 29 PCDD/F congeners have been assigned with toxic equivalency factor (TEF) values relative to that of TeCDD (TEF = 1).^5^ Furthermore, an estimation of PBDD/F risks is recommended for using TEF values of chlorinated analogs.^13^

In addition to PCDD/Fs and PBDD/Fs, mixed polybrominated/chlorinated dibenzo-*p*-dioxins/dibenzofurans (PXDD/Fs, X = Br and Cl) have been detected in indoor dusts, industrial water, waste materials, and fish.^14–17^ In vitro studies have demonstrated that the molecular response profiles induced by PXDD/F exposure are comparable to those of TeCDD, TeBDD, and TeBDF,^18^ suggesting that PXDD/Fs possess in vivo toxicities similar to those of PCDD/Fs and PBDD/Fs. Recently, we found developmental toxicities, such as higher mortality and cardiac abnormalities, in Japanese medaka (*Oryzias latipes*) embryos exposed to 2-chloro-3,7,8-tribromodibenzofuran (TeXDF) and 1,2,3,7,8-pentabromodibenzofuran (PeBDF) detected in industrial water and indoor dusts, respectively.^19^ These toxicities are also exhibited in TeCDD- and TeBDF-exposed embryos of Japanese medaka, implying developmental toxicity of TeXDF and PeBDF in mammals.

Thus, in the present study, we examined ultrasonic vocalizations (USVs) and exploratory behaviors in mouse offspring born to dams exposed to TeXDF and PeBDF because we have revealed abnormalities in these behaviors of mice exposed to TeCDD and TeBDF in utero and via lactation.^20^^,^^21^

## Materials and methods

### Reagents and chemicals

TeXDF and PeBDF (purity >77% each) (Supplementary Fig. S1) were synthesized according to previously described methods.^22^ Corn oil and *n*-nonane were purchased from Wako Pure Chemicals (Osaka, Japan) and Nacalai Tesque (Kyoto, Japan), respectively. The manufacturers of the other reagents and instruments used in this study are described in the corresponding sections.

### Animals and chemical treatment

**Fig. 1:**
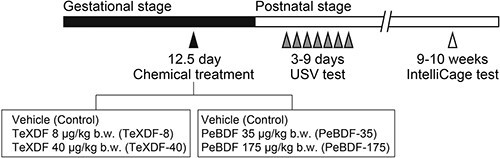
Timeline of chemical treatment and behavioral tests. Pregnant mice were orally treated with vehicle (corn oil containing 0.6% *n*-nonane), TeXDF at a dose of 8 or 40 μg/kg b.w., or PeBDF at a dose of 35 or 175 μg/kg b.w. on GD 12.5. USVs of pups on PNDs 3–9 were recorded, and IntelliCage analysis was performed on 9–10-week old pups.

The animal experiment protocol used in this study was approved by the Animal Use and Care Committee of the National Institute of Environmental Studies (NIES) (No. 23006). The experiments were conducted in strict accordance with NIES guidelines. Pregnant female C57BL/6J mice were purchased from CLEA Japan (Tokyo, Japan) and were housed in an animal facility at a temperature of 24 ± 1 °C and humidity of 50 ± 10%, with a 12/12-h light–dark cycle (lights on from 7:00 to 19:00). Laboratory rodent chow (CE-2) and distilled water were provided ad libitum. To examine the effects of maternal chemical exposure on infant and adult behaviors, pregnant mice were administered using a vehicle (corn oil containing 0.6% *n*-nonane; the control group), TeXDF at a dose of 8 or 40 μg/kg body weight (b.w.) (TeXDF-8 and TeXDF-40 groups, respectively), or PeBDF at doses of 35 or 175 μg/kg b.w. (PeBDF-35 and PeBDF-175 groups, respectively) on gestational day (GD) 12.5 via oral gavage (Fig. 1). All test chemicals were prepared and administered using a vehicle. Infant USV impairments were found in the mouse pups born to dams treated with TeCDD at a dose of 3 μg/kg b.w., but not in those treated with 0.6 μg/kg b.w., on GD 12.5.^21^ Thus, we considered the chemical concentration described above to be equivalent to TeCDD doses of 0.6 or 3.0 μg/kg b.w. on a molar concentration basis according to the fact that the TEF values of 2,3,7,8-tetrachlorodibenzofuran (TeCDF) and 1,2,3,7,8-pentachlorodibenzofuran (PeCDF) are 0.1 and 0.03, respectively.^5^ We ensured that the dams had not given birth until 18:00 on GD 18.5 and used offspring born until 18:00 the following day to perform the experiments. The date of birth was defined as postnatal day (PND) 0.

### USV analysis

Infant USV recordings and analyses were performed as previously described.^20^ Briefly, USVs emitted by mouse pups were recorded using a high-frequency microphone (Type 7116; Aco Co., Tokyo, Japan) and were analyzed using a rodent USV analysis system (Type USV-01; O’Hara Co., Tokyo, Japan). This system was composed of an amplifier (Type 4116, Aco Co.) and a personal computer with installed software (URS-9100 software, O’Hara Co.). The total duration of the USV frequencies of 60–100 kHz was analyzed and compared between the control and exposed groups. On PNDs 3–9, the pups were placed on a hot plate set at 30 °C, and their USVs were recorded in a sound-attenuated chamber for 1 min. The USV recording data were analyzed using UWA-9100 software (O’Hara Co.). To avoid maternal influence, the median values of USV duration and number per litter were compared between the control and exposure groups (30 pups [males: 16, females: 14] from 5 control litters, 30 pups [males: 16, females: 14] from 5 TeXDF-8 litters, and 29 pups [males: 13, females: 16] from 5 TeXDF-40 litters; 44 pups [males: 21, females: 23] from 8 control litters, 41 pups [males: 22, females: 19] from 7 PeBDF-35 litters, and 48 pups [males: 26, females: 22] from 8 PeBDF-175 litters).

### Exploratory behavior analysis

Exploratory behavior in a novel environment was analyzed using IntelliCage, a fully automated testing apparatus for group-housed mice, as previously described.^20^ The IntelliCage consists of a large plastic cage (55 × 37.5 × 20.5 cm) equipped with 4 chambers (15 × 15 × 21 cm each), where mice can drink water by nose-poking. Briefly, male mice aged 9–10 weeks were introduced to IntelliCage, and their spontaneous behaviors were observed for 24 h. At 2–3 days before introduction into the apparatus, the mice were anesthetized with a mixture of medetomidine hydrochloride (Domitol, Meiji Seika Pharma Co., Ltd, Tokyo, Japan), midazolam (Dormicum, Astellas Pharma Inc., Tokyo, Japan), and butorphanol (Vetorphale, Meiji Seika Pharma Inc.). A glass-covered transponder possessing a unique ID code for radiofrequency identification (Datamrs, Temple, TX, United States) was subcutaneously implanted. The antennas recognize a unique ID code at the entrance of each corner. For each mouse, corner visits, nose pokes, and water licking were automatically recorded using the ID code information. Using 2 IntelliCage systems, we simultaneously examined the behavioral patterns of the 32 male offspring. We focused on data from the first day after entering the IntelliCage system when the mice were completely naive to the IntelliCage environment. Since our previous studies have found behavioral changes in male mice perinatally exposed to TeCDD and TeBDF by IntelliCage tests,^20^^,^^23^ the present study used only male offspring of dams exposed to TeXDF and PeBDF to assess the exploratory behavior. The number of animals used was as follows: 11 male mice from 5 control litters, 10 male mice from 5 TeXDF-8 litters, 11 male mice from 5 TeXDF-40 litters, 11 male mice from 8 control litters, 10 male mice from 7 PeBDF-35 litters, and 11 male mice from 8 PeBDF-175 litters.

### Statistical analysis

Statistical analyses were performed using BellCurve for Excel software (Social Survey Research Information Co., Ltd, Tokyo, Japan). USVs (duration and number) and exploratory behaviors (corner visits, nose pokes, and water licking) among the groups were analyzed using 1- or 2-way repeated measures analysis of variance (ANOVA), followed by Tukey–Kramer’s multiple comparison test. A *P* value of <0.05 was considered to be statistically significant in all tests.

## Results

**Fig. 2:**
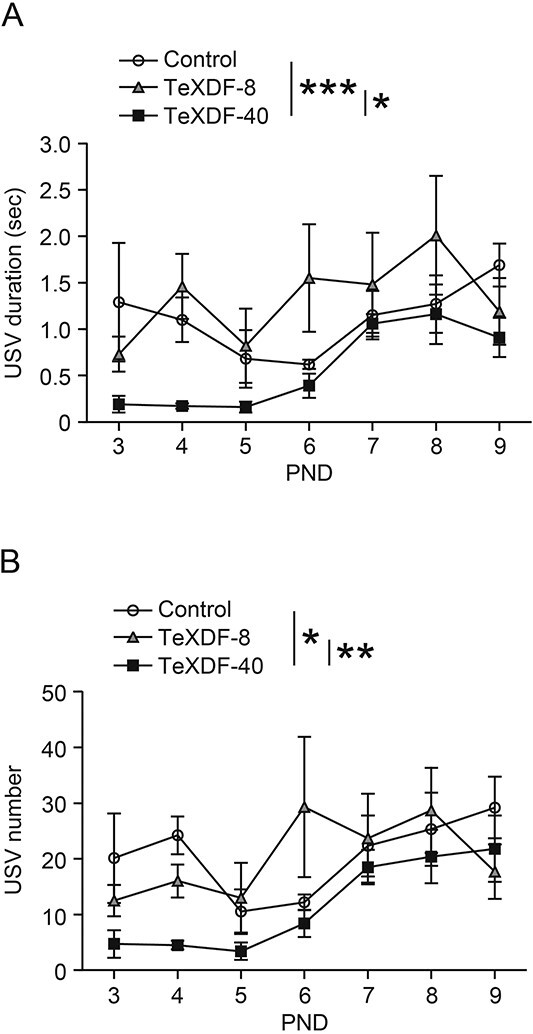
Suppression of USVs emitted by mouse pups of dams exposed to TeXDF. A, B) USV duration (A) and number (B) in the TeXDF-40 group were significantly lower than those in the control and TeXDF-8 groups. The values are shown as the mean ± SEM for 5, 5, and 5 litters (30, 30, and 29 pups from the control, TeXDF-8, and TeXDF-40 groups, respectively). Asterisks (^*^, ^*^^*^, and ^*^^*^^*^) denote statistical differences as determined by the Tukey–Kramer test (*P* < 0.05, <0.01, and <0.001, respectively).

To investigate infant behaviors, we analyzed the USVs of the pups born to dams treated with TeXDF, and we found significant differences in the USV duration and number between the control and TeXDF groups (two-way repeated measures ANOVA; the main effects of postnatal age [*P* < 0.05] and exposure [*P* < 0.01], and interaction between the two main effects [*P* > 0.05], each). The duration in the TeXDF-40 group was significantly lower than that in the control and TeXDF-8 groups (Tukey–Kramer test, *P* < 0.001 and *P* < 0.05, respectively; Fig. 2A). The number of USVs in the TeXDF-40 group was significantly lower than that in the control and TeXDF-8 groups (Tukey–Kramer test, *P* < 0.05 and <0.01, respectively; Fig. 2B). Thus, maternal exposure to TeXDF results in atypical infant behaviors.

**Fig. 3:**
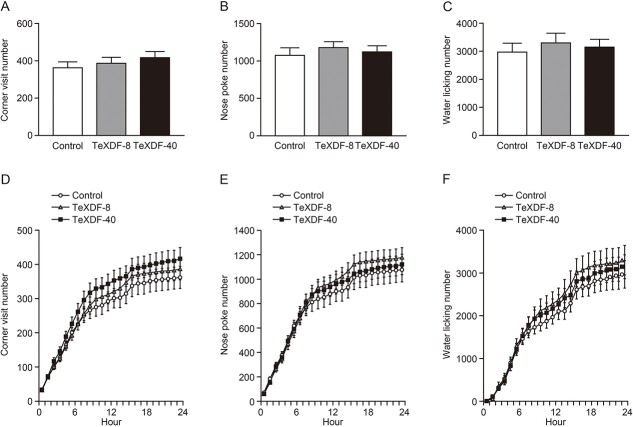
Exploratory behaviors in a novel environment of adult mouse offspring of dams exposed to TeXDF. A–F) The number of corner visits (A, D), nose pokes (B, E), and water licking (C, F). There was no significant difference in the number of the 3 indices for 24 h (A–C) and each hour (D–F) among the groups. The values are the mean ± SEM for 11, 10, and 11 mice from the control, TeXDF-8, and TeXDF-40 groups, respectively.

To examine whether maternal exposure to TeXDF leads to long-lasting behavioral impairments, we analyzed the number of corner visits, nose pokes, and water licking in a novel environment using IntelliCage. There was no significant difference in the 3 indices among the control and exposed groups for 24 h (one-way ANOVA, *P* > 0.05, each; Fig. 3A–C). In addition, we analyzed more detailed behaviors every hour, but no significant change at each hour was found in corner visits, nose pokes, and water licking (one-way ANOVA, *P* > 0.05, each; Fig. 3D–F).

**Fig. 4:**
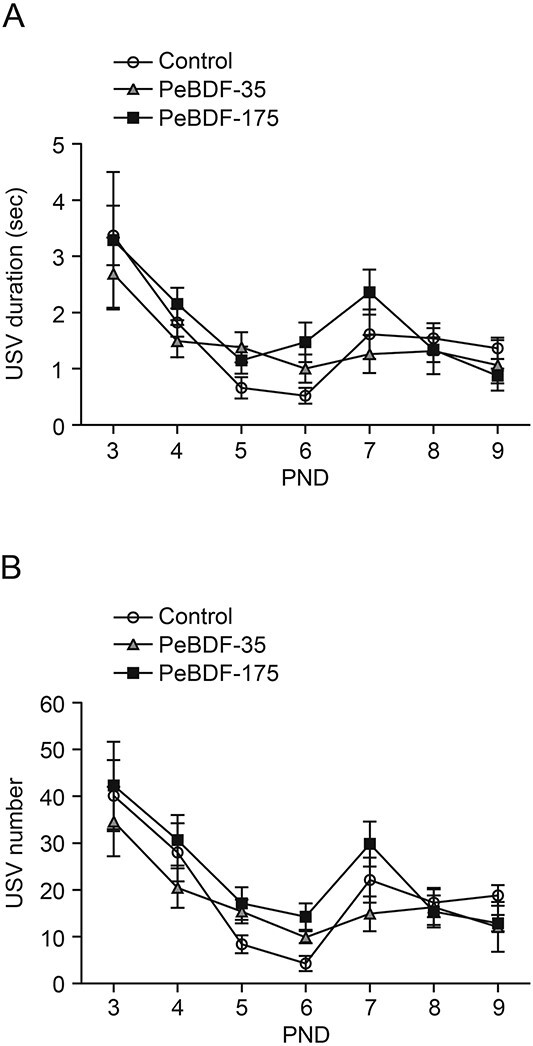
No change in USVs emitted by mouse pups of dams exposed to PeBDF. A, B) USV duration (A) and number (B) were not significantly different among the control, PeBDF-35, and PeBDF-175 groups. The values are the mean ± SEM for 8, 7, and 8 litters (44, 41, and 48 pups from the control, PeBDF-35, and PeBDF-175 groups, respectively).

**Fig. 5:**
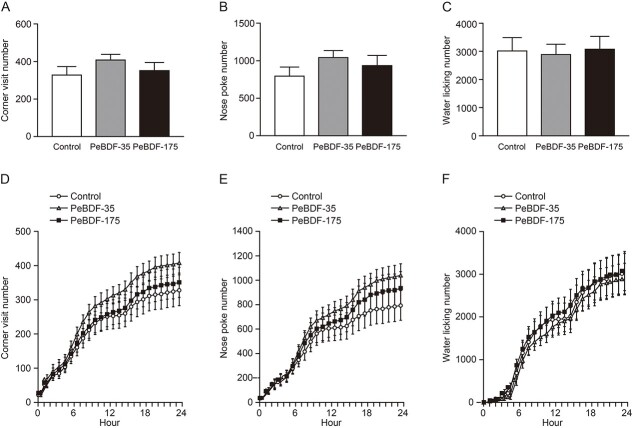
Exploratory behaviors in a novel environment of adult mouse offspring of dams exposed to PeBDF. A–F) The number of corner visits (A, D), nose pokes (B, E), and water licking (C, F). There was no significant difference in the number of the three indices for 24 h (A–C) and each hour (D–F) among the groups. The values are shown as the mean ± SEM for 11, 10, and 11 mice from the control, PeBDF-35, and PeBDF-175 groups, respectively.

While suppressed USVs in infant mice exposed to tetrahalogenated congeners were found in our previous studies^20^^,^^21^ and in the present experiments (Fig. 2A, B), the USV duration and number were not significantly different between the control and PeBDF-exposed groups (two-way repeated measures ANOVA; main effects of postnatal age [*P* < 0.05] and exposure [*P* > 0.05] and interaction between the two main effects [*P* > 0.05]) (Fig. 4A, B). Statistical changes in infant USVs during postnatal development were observed in the groups exposed to PeBDF as well as TeXDF. In the adult offspring, there was no significant change in the number of corner visits, nose pokes, and water licking among the three groups (one-way ANOVA, *P* > 0.05, each; Fig. 5A–F).

## Discussion

Isolated infant mice predominantly communicate by emitting USVs, and abnormalities in infant USVs have been reported in multiple mouse models of neurodevelopmental disorders.^24^ Atypical USV emissions in infant rodents have been shown in developmental neurotoxicity studies focusing on environmental pollutants (Supplementary Table S1). To the best of our knowledge, this study is the first to demonstrate suppressed USVs caused by TeXDF exposure, providing evidence that USVs help to understand developmental neurotoxicity of environmental pollutants.

We could not find any significant difference in the exploratory behavior of adult offspring born to dams exposed to TeXDF, suggesting that the exposure conditions used in the present study did not induce severe neurotoxicity and impaired motor function. However, TeCDD and TeBDD impair other brain functions, such as learning and memory.^7^^,^^8^ Further behavioral tests for these functions are important for the risk assessment of various PXDD/Fs.

Although, in the present study, we did not examine the transfer of TeXDF and PeBDF from dams to their offspring, maternal exposure of PCDD/Fs and PBDD/Fs in utero and via lactation is well known in humans and rodents.^2–4^^,^^6^ Indeed, we found disruption of gene expression in multiple organs, including the brain and liver, of mouse pups born to dams administered with tetrahalogenated congeners (i.e. TeCDD and TeBDF),^25^^,^^26^ supporting the idea that the pups exhibited atypical USVs following in utero and lactational exposure to TeXDF.

Based on the carbon skeleton and position of the halogen substitution, the TEF values of TeCDF (0.1) and PeCDF (0.03) were adapted for TeXDF and PeBDF, respectively.^13^ Suppressed USVs were observed in infant mouse pups of dams administered TeCDD (TEF = 1) at a dose of 3 μg/kg b.w.^21^ We used TeXDF and PeBDF doses of 40 and 175 μg/kg b.w., respectively, equivalent to a TeCDD dose of 3 μg/kg b.w. on a molar concentration basis. The pups of TeXDF-exposed dams exhibited a decrease in USV duration and number (Fig. 2A, B), supporting its developmental neurotoxicity in accordance with a TEF value of 0.1. Intriguingly, no significant change in infant USVs was found in the case of PeBDF exposure, suggesting that the TEF value was <0.03. Although we do not have any experimental data to explain the results, one possibility is the difference in gestational absorption between PeBDF and 2,3,7,8-substituted congeners. In the PCDD/F group, the amount of PeCDF absorbed was lower than that of TeCDD and TeCDF in rat organs.^27^ The uptake of TeXDF was similar to that of TeCDD in Japanese medaka, while PeBDF showed a lower uptake rate.^19^ Accordingly, in the present study, it is plausible that the transfer of PeBDF from dams to pups in utero and via lactation might not be sufficient for inducing atypical USVs. Biochemical and molecular analyses, such as chemical concentration, gene expression, and enzymatic activity, in the organs of exposed pups will help to determine more accurate TEF values for PBDD/Fs and PXDD/Fs.

## Conclusion

Our findings highlight the importance of biological studies to evaluate the developmental toxicity and risk assessment of PXDD/Fs. In particular, the results of this study support the idea that infant USVs are valuable for understanding the developmental neurotoxicity of a wide range of environmental pollutants. The toxic effect of PXDD/Fs warrants further investigation of the molecular mechanisms underlying perinatal exposure to behavioral abnormalities.

## Supplementary Material

Proof_230816_Suppl_PDF_tfad069

## Data Availability

The datasets used and analyzed during this study are available from the corresponding author on reasonable request.
