# Supplementary material for: 2-Chloro-3,7,8-tribromodibenzofuran as a new environmental pollutant inducing atypical ultrasonic vocalization in infant mice
Source: Toxicol Res (Camb). 2023 Aug 23;12(5):999–1004. doi: 10.1093/toxres/tfad069 (PMC10615804; doi:10.1093/toxres/tfad069)
Supplement: Proof_230816_Suppl_PDF_tfad069 [file proof_230816_suppl_pdf_tfad069.pdf]

Supplementary material

2-Chloro-3,7,8-tribromodibenzofuran as a new environmental pollutant inducing atypical ultrasonic vocalization in infant mice

Eiki Kimura[1,2,3], Go Suzuki[4], Naoto Uramaru[5], Masaki Kakeyama[6], Fumihiko Maekawa[1,*]

[1] Health and Environmental Risk Research Division, National Institute for Environmental Studies, Tsukuba, 305-8506 Japan

[2] Japan Society for the Promotion of Science, Tokyo, 102-0083 Japan

[3] Department of Environmental Health, University of Fukui School of Medical Sciences, Fukui, 910-1193 Japan

[4] Material Cycles Division, National Institute for Environmental Studies, Tsukuba, 305-8506 Japan

[5] Division of Pharmaceutical Helath Biosciences, Nihon Pharmaceutical University, Saitama, 362-0806 Japan

[6] Faculty of Human Sciences, Waseda University, Saitama, 359-1192 Japan

*** Corresponding author**

Fumihiko Maekawa, Health and Environmental Risk Research Division, National Institute for Environmental Studies, 16-2 Onogawa, Tsukuba 305-8506, Japan. Tel: +81-29-850-2402. Email: fmaekawa@nies.go.jp

Table S1. Environmental pollutants affecting ultrasonic vocalizations in infant rodents.

Chemicals	Species (strains)	Toxic effects	References
**Metals**			
Methyl mercury	Rat (Sprague-Dawley)	Increased duration<br>Decreased max. and min. frequency	[1]
	Rat (Wistar)	Decreased duration and number	[2]
Lead	Rat (Wistar)	Decreased number (7 days old)<br>Increased number (14 days old)	[3]
Aluminium	Mouse (CBA/T6)	Decreased number	[4]
**Pesticides**			
Chlorpyrifos	Rat (Sprague-Dawley)	Decreased number	[5]
	Rat (Wistar)	Decreased number<br>Longer latency to the first call	[6]
	Mouse (CD-1)	Decreased duration and number<br>Increased peak frequency	[7]
	Mouse (C57BL/6)	Decreased duration and number	[8]
**Herbicides**			
Glyphosate-based herbicide	Rat (Wistar)	Decreased number	[9]
**Polychlorinated biphenyls (PCBs)**			
PCB 47 and PCB 77	Rat (Sprague-Dawley)	Decreased number	[10]
Aroclor 1221 (PCB mixture)	Rat (Sprague-Dawley)	Decreased number	[11]
**Polychlorinated dibenzo-*p*-dioxins/dibenzofurans**			
2,3,7,8-Tetrachlorodibenzo-*p*-dioxin	Mouse (C57BL/6)	Decreased duration<br>Decreased complicated call types	[12]
**Polybrominated dibenzo-*p*-dioxins/dibenzofurans**			
2,3,7,8-Tetrabromodibenzofuran	Mouse (C57BL/6)	Decreased duration and number	[13]
**Mixed polybrominated/chlorinated dibenzo-*p*-dioxins/dibenzofurans**			
2-Chloro-3,7,8-tribromodibenzofuran	Mouse (C57BL/6)	Decreased duration and number	Present study

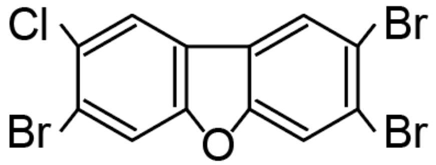

2-Chloro-3,7,8-tribromodibenzofuran
(TeXDF)
MW = 439
Interim TEF value = 0.1

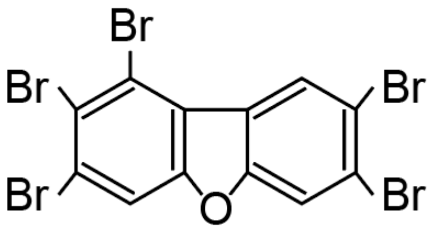

1,2,3,7,8-Pentabromodibenzofuran
(PeBDF)
MW = 563
Interim TEF value = 0.03

Figure S1. The chemical structure and molecular weights of 2-chloro-3,7,8-tribromodibenzofuran (TeXDF) and 1,2,3,7,8-pentabromodibenzofuran (PeBDF).

References

1. Cagiano R. Cortese I., De Salvia M. A., Renna G., Cuomo V., Effects of prenatal exposure to methyl mercury on ultrasonic calling in rat pups, *Pharmacol. Res. Communivations*, 1988, 20, 215–216.
2. Elsner J., Suter D., Alder S., Microanalysis of ultrasound vocalizations of young rats: assessment of the behavioral teratogenicity of methylmercury, *Neurotoxicol. Teratol.*, 1990, 12, 7–14.
3. De Marco M., Halpern R., Barros H. M., Early behavioral effects of lead perinatal exposure in rat pups, *Toxicology*, 2005, 211, 49–58.
4. Rankin J., Manning A., Alterations to the pattern of ultrasonic calling after prenatal exposure to aluminium sulfate, *Behav. Neural Biol.*, 1993, 59, 136–142.
5. Berg E. L., Ching T. M., Bruun D. A., Rivera J. K., Careaga M., Ellegood J., Lerch J. P., Wöhr M., Lein P. J., Silverman J. L.,Translational outcomes relevant to neurodevelopmental disorders following early life exposure of rats to chlorpyrifos, *J. Neurodev. Disord.*, 2020, 12, 40.
6. Morales-Navas M., Castaño-Castaño S., Pérez-Fernández C., Sánchez-Gil A., Teresa Colomina M., Leinekugel X., Sánchez-Santed F., Similarities between the effects of prenatal chlorpyrifos and valproic acid on ultrasonic vocalization in infant Wistar rats, *Int. J. Environ. Res. Public Health*, 2020, 17.
7. Venerosi A., Ricceri L., Scattoni M. L., Calamandrei G., Prenatal chlorpyrifos exposure alters motor behavior and ultrasonic vocalization in CD-1 mouse pups, *Environ. Health*, 2009, 8, 12.
8. Mullen B. R., Khialeeva E., Hoffman D. B., Ghiani C. A., Carpenter E. M., Decreased reelin expression and organophosphate pesticide exposure alters mouse behaviour and brain morphology, *ASN Neuro*, 2012, 5, e00106.
9. de Oliveira M. A. L., Rojas V. C. T., de Sá J. C., de Novais C. O., Silva M. S., de Almeida Paula H. A., Kirsten T. B., Bernardi M. M., Pinheiro L. C., Giusti-Paiva A., and Vilela F. C., Perinatal exposure to glyphosate-based herbicides induced neurodevelopmental behaviors impairments and increased oxidative stress in the prefrontal cortex and hippocampus in offspring, *Int. J. Dev. Neurosci.*, 2022, 82, 528–538.
10. Krishnan D. C., Cromwell H. C., Meserve L., Effects of polychlorinated biphenyl (PCB) exposure on response perseveration and ultrasonic vocalization emission in rat during development, *Endocr. Disruptors*, 2014, 2, e969608.
11. Krishnan K., Rahman S., Hasbum A., Morales D., Thompson L. M., Crews D., Gore A. C., Maternal care modulates transgenerational effects of endocrine-disrupting chemicals on offspring pup vocalizations and adult behaviors, *Horm. Behav.*, 2019, 107, 96–109.

12. Kimura E., Tohyama C., Vocalization as a novel endpoint of atypical attachment behavior in 2,3,7,8-tetrachlorodibenzo-p-dioxin-exposed infant mice, *Arch. Toxicol.*, 2018, 92, 1741–1749.
13. Kimura E., Suzuki G., Uramaru N., Endo T., Maekawa F., Behavioral impairments in infant and adult mouse offspring exposed to 2,3,7,8-tetrabromodibenzofuran in utero and via lactation, *Environ. Int.*, 2020, 142, 105833.
